# Modeling Chemical Reactivity at the Interfaces of Emulsions: Effects of Partitioning and Temperature

**DOI:** 10.3390/molecules26154703

**Published:** 2021-08-03

**Authors:** Marlene Costa, Fátima Paiva-Martins, Sonia Losada-Barreiro, Carlos Bravo-Díaz

**Affiliations:** 1 REQUIMTE/LAQV, Department of Chemistry and Biochemistry, Faculty of Science, University of Porto, Rua do Campo Alegre 687, 4169-007 Porto, Portugal; marlene.andreia.costa@gmail.com (M.C.); mpmartin@fc.up.pt (F.P.-M.); 2 Departamento de Química—Física, Facultad de Química, Universidade de Vigo, 36310 Vigo, Spain; sonia@uvigo.es

**Keywords:** kinetics, pseudophase model, emulsions, antioxidants

## Abstract

Bulk phase chemistry is hardly ever a reasonable approximation to interpret chemical reactivity in compartmentalized systems, because multiphasic systems may alter the course of chemical reactions by modifying the local concentrations and orientations of reactants and by modifying their physical properties (acid-base equilibria, redox potentials, etc.), making them—or inducing them—to react in a selective manner. Exploiting multiphasic systems as beneficial reaction media requires an understanding of their effects on chemical reactivity. Chemical reactions in multiphasic systems follow the same laws as in bulk solution, and the measured or observed rate constant of bimolecular reactions can be expressed, under dynamic equilibrium conditions, in terms of the product of the rate constant and of the concentrations of reactants. In emulsions, reactants distribute between the oil, water, and interfacial regions according to their polarity. However, determining the distributions of reactive components in intact emulsions is arduous because it is physically impossible to separate the interfacial region from the oil and aqueous ones without disrupting the existing equilibria and, therefore, need to be determined in the intact emulsions. The challenge is, thus, to develop models to correctly interpret chemical reactivity. Here, we will review the application of the pseudophase kinetic model to emulsions, which allows us to model chemical reactivity under a variety of experimental conditions and, by carrying out an appropriate kinetic analysis, will provide important kineticparameters.

## 1. Introduction

The three-dimensional interfaces between two immiscible liquids (oil and water) found in emulsions and other colloidal systems have been widely used as models of membrane function to mimic their behavior, as models of lipid-based foods and to model important (bio)chemical reactions such as the oxidation of lipids [1,2,3,4]. A variety of fundamental processes involved in biocatalysis, such as ion pumping, electron transport, membrane fusion, and photosynthesis, have all been investigated in such interfacial systems for further guidance in developing new biomaterials and medicines and to better understand life science [5,6,7,8,9,10,11].

**Figure 1:**
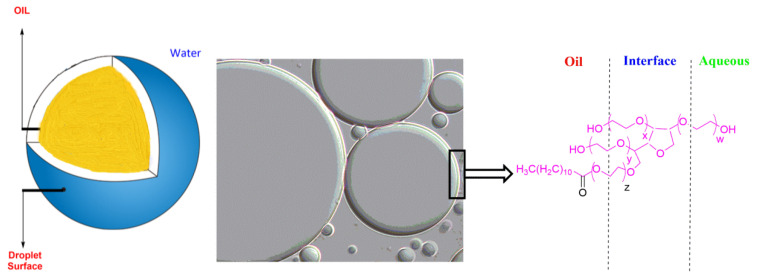
(**Left**) Basic illustration of an oil-in-water emulsion droplet. The diameter of the droplet may range from tens of nanometers to micrometers, and the volume of the interfacial region is only a small fraction of the total volume of the droplet. (**Right**) Optical microscope photograph of an O/W emulsion stabilized with Tween 20 and conceptual division of the domains with different solvent properties.

Emulsions are composed of a liquid dispersed as small droplets in a second immiscible liquid. The droplet interface is encompassed by a narrow region (2–20 nm thick) surrounding each emulsion droplet, Figure 1 [12]. The interfacial region typically does not contribute significantly to the total volume of an emulsion unless the droplet sizes are very small [13], but has important consequences in the fate of chemical reactions that take place in the emulsions. For example, lipid oxidation reactions and those between antioxidants and lipid radicals are largely affected by the presence of interfacial regions, because they are highly anisotropic regions composed of a mélange of oil, water, and surfactants [2,14,15,16].

Conceptually, emulsions can be divided into three regions with distinct solvent properties: the core of droplets, the continuous phase, and the interfacial region (Figure 1). The interfacial layer surrounding each emulsion droplet has a crucial role in the applicability and properties of emulsions because many important chemical, biological, physical, and technological processes take place at interfaces [16,17,18,19]. Reactive components can partition between the different regions, reducing or increasing the concentrations of these components in the site of reaction. The differential solubility of reactants (and of reaction products) in the various regions of the system can alter their local concentrations, making possible to carry out reactions at rates much higher (or much lower) than those in bulk homogeneous phases, allowing the tuning of reaction rates. It is not strange, therefore, that the interfaces between two immiscible liquids can serve as convenient models for investigating the fate of chemical reactions in more complex natural systems such as cell membranes [20,21,22,23].

To interpret chemical reactivity in emulsions, a kinetic model based on the formalism of the pseudo-phase was devised [19,24,25]. In this article, we will summarize its main features and applications. We will also show some illustrative examples that highlight the importance of reactant partitioning and of the main outcomes of the model, for example, determining activation parameters of reactions taking place in the interfacial region of emulsions. The model was originally developed aiming at solving an important, but refractory, problem in food science: how to quantify the effective concentration of antioxidants added to slow lipid oxidation in the oil, water, and interfacial regions of food grade, lipid-based emulsions.

In the pseudo-phase model, the whole solution is divided into oil, interfacial, and aqueous regions (pseudo-phases), with the surfactant located in the boundary between the oil and water regions [19,24,26,27,28,29,30]. Each region has different solvent properties and is treated as a separate phase or pseudo-phase. Reactants (and other components) partition between the regions thermodynamically, depending on the values of the Gibbs free energy of transfer. This implies that the distribution of the reagents is always described by partition constants whose value is the same throughout the course of the reaction.

However, some complications may arise when considering thick emulsions where transport of reactants between regions can be rate-limiting [13,31]. For the sake of simplicity, the review will focus exclusively on fluid emulsions where restrictions to the free movement of reactants and other components, if any, do not affect molecular transport. Prior to considering modeling the chemical reactivity, we will briefly discuss some properties of emulsions and their dynamic aspects.

## 2. Emulsions: Classification, Physical Stabilization, and Main Properties

**Scheme 1:**
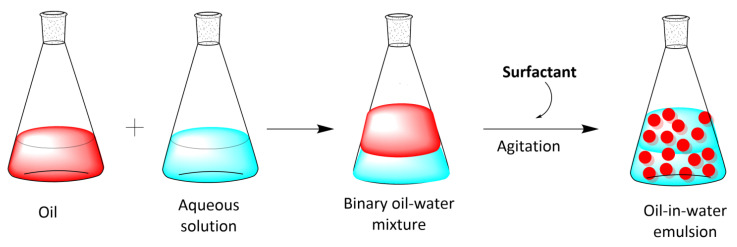
Formation of emulsions after mixing oil, an aqueous solution, and the appropriate surfactant with some agitation. Addition of surfactants is needed to stabilize kinetically the droplets.

Emulsions are mixtures of two immiscible liquids such as oil and water, where one of the liquids is dispersed as small spherical droplets in the other one. They can be prepared, Scheme 1, by either shaking together the oil and water or by adding one of the liquids drop by drop to the other liquid with agitation. However, the surface of each droplet is an interface between hydrophilic and hydrophobic molecules. The contact between the molecules of the two immiscible liquids is highly unfavorable because of the increased enthalpy caused by the contact between hydrophilic and hydrophobic molecules, which is not compensated by the increased entropy of mixing. As a result, emulsions are thermodynamically unstable colloidal systems, which tend to separate the two liquids until the contact area between them is the lowest possible (i.e., minimal Gibbs free energy).

**Scheme 2:**
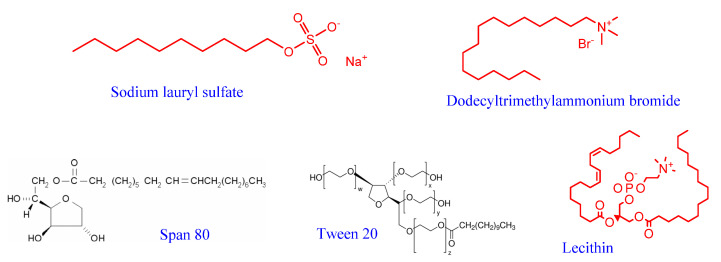
Chemical structures of some common surfactants employed to stabilize kinetically emulsions.

To stabilize kinetically emulsions, surfactant molecules are added to the binary oil–water system. Surfactants are amphiphilic molecules having both hydrophilic, water-soluble and lipophilic, oil soluble residues that interact simultaneously with the oil and water, so that they locate at the droplet surface with their hydrophilic part of the emulsifier pointing to the water and its lipophilic part pointing to the oil, forming a three-dimensional boundary surrounding the surface of the droplets, Figure 1. This arrangement makes emulsions be stabilized kinetically during periods of time ranging from minutes to years, depending on the composition of the emulsion. Thus, an appropriate choice of the surfactant is crucial in the formation of the emulsion and its long-term stability. Surfactants can be anionic, cationic, non-ionic, and zwitterionic, Scheme 2, and they play a major role on the formation, stability, and functional characteristics of the emulsion.

A valuable tool for classifying surfactants and their main applications involves the so-called hydrophilic lipophilic balance (HLB) value. The HLB of an emulsifier represents a balance between the hydrophilic (polar) portion of the surfactant and the lipophilic (non-polar) groups of the emulsifier. The more lipophilic surfactants have low HLB values (<9.0); meanwhile, the more hydrophilic ones have a high HLB number (>11.0). When two or more surfactants are blended, the resulting HLB of the blend is easily calculated taking into consideration the HLB of each surfactant and its relative proportion. Table 1 illustrates the range of HLB values that are most suited for a particular application.

**Figure 2:**
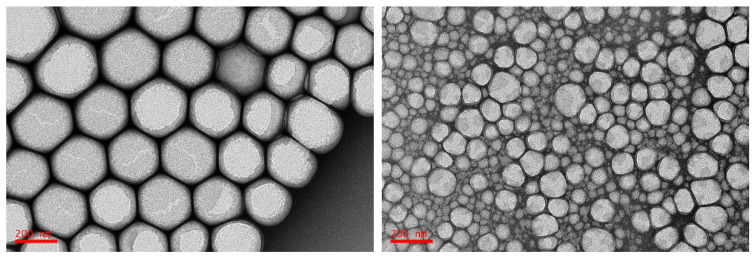
TEM microphotographs (15,000×) of stained (uranyl acetate) of 1:9 (*vol*:*vol*) fish oil-in-water nanoemulsions prepared with different amounts of surfactant. (**Left**) Φ_I_ = V_surf_/V_emulsion_ = 0.0047, (**right**), Φ_I_ = 0.0378. Reproduced from ref. [34].

Emulsions can be conveniently classified in terms of the relative organization of the oil and aqueous phases. When oil droplets are dispersed in the aqueous phase, oil-in-water (O/W) emulsions are formed. On the contrary, when the aqueous phase is dispersed in the oil, water-in-oil (W/O) emulsions are formed. Multiple emulsions (W/O/W and O/W/O systems) are described as an emulsion of an emulsion. For example, a water-in-oil-in-water (W/O/W) emulsion consists of water droplets dispersed within oil droplets, which are themselves dispersed in an aqueous phase. Emulsions can also be classified according to the size of the droplets as macroemulsions (or, simply, emulsions), characterized by droplet size ranging from 0.1 to 5 µm, or nanoemulsions (droplets in the range of 20–100 nm). The microstructure of the droplets can be visualized by employing X-ray transmission electron microscope (TEM) as illustrated in Figure 2. Droplets are approximately spherical in morphology and their sizes, typically ranging 70–2000 nm, depending on various factors, including the surfactant volume fraction employed in the preparation of the emulsion, the energy input employed, the nature of the oil and surfactant, and temperature.

**Scheme 3:**
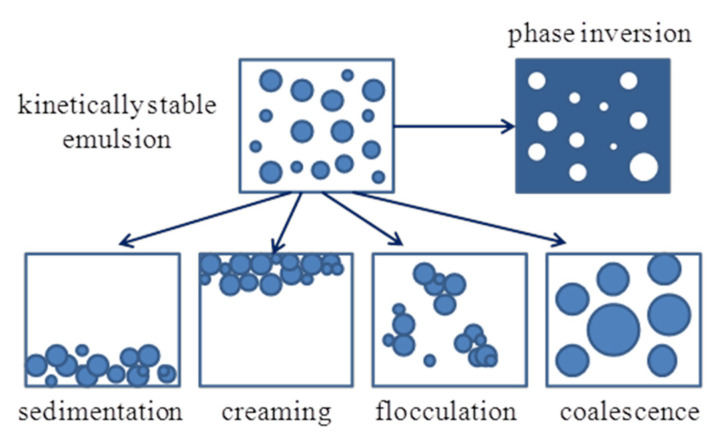
Main destabilization mechanisms of emulsions. Adapted from ref. [36].

Emulsions are thermodynamically unstable systems and tend to phase separation with time. Scheme 3 shows the main emulsion destabilization mechanisms of emulsions and nanoemulsions. Nanoemulsions are, in general, more resistant to physical destabilization via creaming, sedimentation, coalescence, and/or flocculation than those with larger droplets, in part, because of the efficient steric stabilization provided by the surfactant [35]. In addition, Brownian motion usually overcomes gravitational forces in emulsions with small droplets. On the contrary, nanoemulsions are mostly susceptible to destabilization through the Ostwald ripening mechanism [9,32,35].

Conceptually, emulsions can be divided into three regions with distinct solvent properties: the core of droplets, the continuous phase, and the interfacial region (Figure 1). The interfacial layer surrounding each emulsion droplet has a crucial role in the applicability and properties of emulsions because many important chemical, biological, physical, and technological processes take place at interfaces [16,17,18,19]. The properties of the oil droplet surface control, to a large extent, the physical stability of emulsions. The nature and concentration of surfactants, together with the composition of the aqueous phase (pH, ionic strength, and ions) determine the electrostatic charge and the thickness of the interfacial layer, both parameters being deeply involved in the physical stability of emulsions [13]. However, the interfacial layer also has an important role in the fate of chemical reactions because reactants in emulsions are compartmentalized [19]. Polar molecules distribute preferentially, between the aqueous and interfacial regions; meanwhile, nonpolar molecules distribute preferentially between the oil and interfacial regions. Molecules of moderate hydrophobicity are distributed between the three regions and those that are both oil and water insoluble are exclusively located in the interfacial region.

## 3. Dynamic Aspects of Emulsions and Mass Transfer

Emulsions are highly dynamic systems and diffusion within droplets, exchange of material between droplets, and self-diffusion of droplets in the continuous phase are common dynamic processes having different time scales, which may affect their potential use [31,37,38,39,40]. Molecular diffusion in emulsions has attracted considerable interest to control the macroscopic physicochemical properties of the system. For example, Ostwald ripening involves the transfer of oil through the aqueous phase, while coalescence involves oil transfer achieved by fusion of the emulsifier film (a droplet–droplet direct interaction) [41]. Interfacial adsorbed surfactants are also dynamic, experiencing lateral and transversal diffusion processes, undergoing continuous reorganization, and are transferred between emulsion droplets [20,42,43]. Surfactants are able to form micelles in the water phase, and they may form swollen micelles (which are several nm in size) that contain oil molecules. These swollen micelles may carry small fractions of oil from small to big droplets and may contribute to the ripening of the emulsions.

Molecular diffusion in emulsions is also relevant to control the kinetic processes of the various chemical reactions that take place at the liquid–liquid interfacial boundaries because emulsion droplets are broken and reorganized constantly, being oil, surfactants, and other emulsion components (rapidly) transferred between droplets [39,44,45]. Diffusion of reactive molecules within or between emulsion droplets depends not only on their molecular nature but also on the particular properties of the components of the emulsions (composition, polarity and viscosity of the oil, components of the water phase, nature of the emulsifier, etc.). Diffusion can be either faster or slower than the undergoing chemical reaction. If the observed rate depends on the rate of molecular diffusion, the reactions are said to be under microscopic diffusion control; meanwhile, if the observed rate depends on the rate at which solutions mix, they are said to be macroscopic diffusion control. When the reaction is slow in comparison with the emulsion dynamics, the reaction “sees” the emulsion droplets as static entities, so that the observed rate of the reaction can be computed as the sum of the rates in all regions of the emulsion. On the contrary, when the diffusion of reactants is rate-limiting, the dynamics of the droplets needs to be taken into account to explain the fate of the chemical reactions.

### 3.1. Kinetics of Chemical Reactions at Interfaces: Diffusive vs. Reactive Systems

Most kinetic theories have been mainly developed to describe the fate of reactions taking place in homogeneous systems, where ideal conditions are usually assumed. However, emulsions are complex systems containing oils, aqueous solutions (where ionic and nonionic compounds can be dissolved in different extents), and an interfacial region connecting the oil and water phases created upon addition of surfactants. In emulsions, several processes occurring on a variety of time scales take place simultaneously. For example, emulsion droplets break and reform continuously, and emulsion components such as oil, water, surfactants, antioxidants, lipid radicals, or other reactive molecules exchange between droplets. Thus, the rate of the chemical reactions that takes place at the interfacial region may depend on the rate of transport of emulsion components between the three regions of the emulsion and eventually may be rate-limiting [19,24,37]. 

To predict whether the dynamics of the droplets affect the chemical reaction, a practical approach, based on the Smoluchowski theory for Brownian motion [46] and the application of the Acree–Curtin–Hammet principle [47], was developed by Bravo et al. [48]. The approach only considers “ideal” systems, where there are no physical barriers limiting the transport of molecules between the oil, aqueous, and interfacial regions and, therefore, where there are no significant deviations from simple diffusion laws, molecular mobility, partitioning, and volume exclusion effects, etc. [48].

**Scheme 4:**
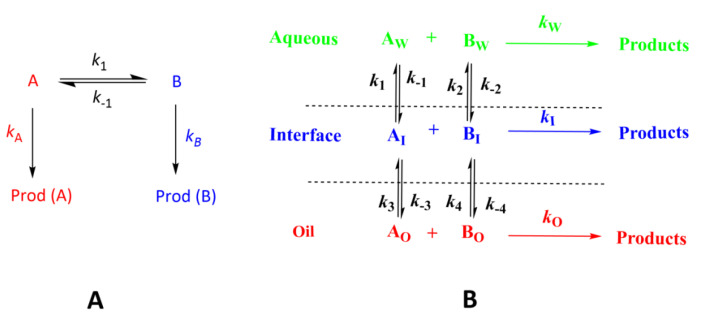
(**A**) Illustrative representation of the formation of different products from two reactants in equilibrium with one another, such as stereoisomers, constitutional isomers, conformational isomers, etc., showing the rate constants involved. (**B**) Illustrative representation of a bimolecular reaction taking place in an emulsion where the reactants (**A**,**B**) can distribute and react simultaneously in the three regions.

The Acree–Curtin–Hammett (ACH) principle [47,49] is a well-known concept described in many physical organic and kinetics textbooks commonly used to explain the selectivity ratios for stereoselective reactions such as that depicted in Scheme 4A. Briefly, the ACH principle states that: “*if the magnitude of the rates involving the equilibrium process of A and B (k1, k − 1) are much higher than those rates of the chemical reactions leading to the formation of products (kA, kB), then the system is under kinetic control, and the final product ratio is equal to the ratio of the product forming rate constants, Equation (1), where K is the equilibrium constant (K = k1/k − 1)*”. On the contrary, if the rates of diffusion are lower than the rates of the chemical reaction, then the reaction is diffusion-controlled and the product distribution depends, in a complex manner, on both the relative size of the diffusion rate constants and the chemical rate constants.

(1) $$\frac{{\left[P_{A}\right]}_{\infty }}{{\left[P_{A}\right]}_{\infty }}=\frac{k_{A}}{k_{B}}\frac{1}{K}$$

The Acree–Curtin–Hammett ideas can be extended to settings where the reactants partition between regions of different solvent properties as depicted in Scheme 4B [19,48]. Only when the rates of transport of molecules (i.e., the rates of diffusion) between regions (*k*1, *k* − 1, *k*2, *k* − 2, *k*3, *k* − 3, *k*4, *k* − 4) are much faster than the rates of the chemical reactions (*kW*, *kI*, *kO*), the overall rate of the reaction is the sum of the rates of the reactions in every phase. Under these conditions, the system is said to be in “dynamic equilibrium” throughout the course of the chemical reaction. On the contrary, when the diffusion of reactants is rate-limiting, the dynamics of the droplets needs to be taken into account to explain the fate of the chemical reactions [50,51,52,53], and the product distribution depends, in a complex manner, on both the relative size of the diffusion rate constants and the chemical rate constants.

A simple mathematical model, grounded on the transition state theory (TST) and diffusion laws, permits us to analyze the conditions required to distinguish between kinetically controlled and diffusion-controlled reactive systems based on molecular and solvent properties. For the sake of clarity, only the main assumptions and conclusions of the model will be presented and the interested reader is referred to specialized publications [46,48,50,51].

The model considers two neutral reactive, spherical, molecules A and B, having radii *r*_A_ and *r*_B_, respectively, in a fluid of a given viscosity (for example, in water) with an energy enough to overcome the activation barrier. The model assumes that the observed rate does not depend on the rate at which the reactants are mixed (i.e., mixing effects are unimportant), that reactants are uniformly distributed, and that the orientation and steric effects are unimportant. Therefore, the estimated rate constants will be an upper limit because if any of the assumptions does not apply, then the true rate constant will be a fraction of that estimated.

If the reactive molecules react immediately when their distance is equal to, or less than, the sum of their radii (i.e., upon an encounter), the reaction is under full microscopic diffusion control, and an approximate value for the rate of the reaction between neutral molecules can be found by employing Fick´s law of diffusion. Since the reactive molecules are usually much larger in size than the solvent molecules, Stoke’s law, Equation (2), applies, and Equation (3) can be derived [50,54]. In Equations (2) and (3), *D* is the diffusion coefficient, *k*_B_ is the Boltzmann’s constant, *T* is the temperature, *η* is the dynamic viscosity of the media, and *r* the hydrodynamic radius of the reactive molecules.

(2) $$D=\frac{k_{B}T}{6\pi \eta r}$$

(3) $$k_{D}=\frac{2k_{B}T}{3\eta }\left(\frac{{\left(r_{A}+r_{B}\right)}^{2}}{r_{A}r_{B}}\right)$$

In water, at *T* = 25 °C, the viscosity is *η* ~ 0.9 cP s, and for molecules of similar size (*r*_A_ ≈ *r*_B_), a value of *k*_D_ ≈ 7 × 10^9^ dm^3^ mol^−1^ s^−1^ can be estimated for the bimolecular reaction. This *k*_D_ value represents an upper limit value for diffusion-controlled reactions of neutral molecules. That is, neutral molecules cannot react faster than the rate at which they diffuse in the solvent. If reactive ions are involved, the value is expected to be somewhat higher because the electrostatic interactions increase their diffusivity. For example, values of *k*_D_ ranging (3 − 15) × 10^10^ dm^3^ mol^−1^ s^−1^ have been estimated for the reaction between hydrogen ions and various anions [50].

If the chemical process is not fast enough to remove the reactive molecules completely as they reach the critical distance *r*_AB_, then the observed rate constant, *k*_obs_, depends on the relative values of *k*_D_ and the rate constant of the chemical process, *k*_chem_, Equation (4). Note that Equation (4) reduces to Equation (3) when *k*_chem_ >>> 4*π*(*D*_A_ + *D*_B_)*r*_AB_; meanwhile, when *k*_chem_ <<< 4*π*(*D*_A_ + *D*_B_)*r*_AB_, *k*_obs_ = *k*_chem_ (i.e., no diffusion control).

(4) $$k_{\mathrm{obs}}=\frac{k_{\mathrm{chem}}}{1+\frac{k_{\mathrm{chem}}}{4\pi (D_{A}+D_{B})r_{\mathrm{AB}}}}=\frac{k_{\mathrm{chem}}}{1+\frac{k_{\mathrm{chem}}}{k_{D}}}$$

**Figure 3:**
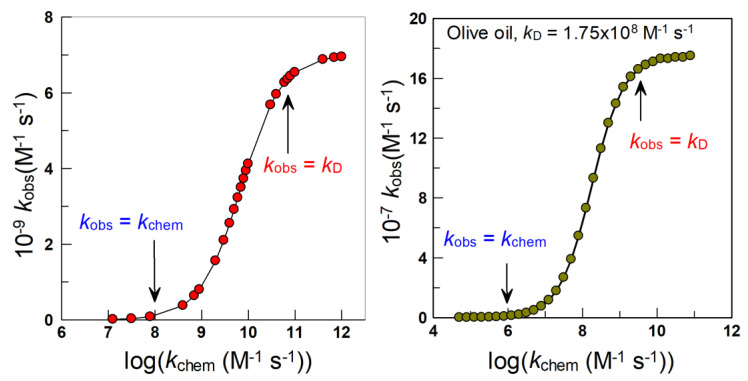
Changes in *k*_obs_ as a function of log(*k*_chem_) for a bimolecular reaction in aqueous solution, according to Equation (4). Figures extracted from ref. [48].

An analysis of the variation of *k*_obs_ with *k*_chem_ for a given value of *k*_D_, Equation (4), allows us to predict if the observed rate constant is, or is not, affected by the diffusion of reactants and in what extent it is. For the purpose, we can plot the variation of *k*_obs_ with *k*_chem_ for a given upper limit value of *k*_D_. Thus, by setting the previously estimated value of *k*_D_ = 7 × 10^9^ dm^3^ mol^−1^ s^−1^ (*T* = 25 °C) for a bimolecular reaction in aqueous solution, the variations of *k*_obs_ with log(*k*_chem_), Figure 3, can be determined. As shown, diffusion-controlled reactions (*k*_obs_ ≈ *k*_D_) are those whose rate of the chemical reaction is *k*_chem_ ≥ 10^12^ M^−1^ s^−1^. Therefore, for kinetically controlled reactions, *k*_obs_ ≈ *k*_chem_ and *k*_chem_ should be <10^9^ dm^3^ mol^−1^ s^−1^. For intermediate *k*_chem_ values (10^9^–10^11^ dm^3^ mol^−1^ s^−1^), the reaction is controlled by both diffusion and activation in a different extent, depending on the particular *k*_chem_ value. For example, when *k*_chem_ ~ *k*_D_ (=7 × 10^9^ dm^3^ mol^−1^ s^−1^ ), *k*_obs_ ≈ 3 × 10^9^, i.e., approximately 50% of diffusion control.

If the same reaction takes place in a more viscous fluid such as olive oil, the upper value for *k*_D_ will be ≈2 × 10^8^ dm^3^ mol^−1^ s^−1^, and only reactions with *k*_chem_ < 10^6^ dm^3^ mol^−1^ s^−1^ will be kinetically controlled [48].

The model also considers the role of the activation energy of the chemical reaction that, according to the TST theory, largely affects the chemical reaction rate. Comparison of the *k_chem_* values in aqueous solution, Table 2, with the diffusion rate values estimated in Figure 3 indicates that for any chemical reaction with *E_a_* higher than ~25 kJ mol^−1^, *k*_obs_ = *k*_chem_, i.e., for most thermally activated reactions that may take pace in emulsions, the reactants are distributed according to their solubilities in the different regions of the emulsion before they react, and the rates of diffusion of the reactants are much higher than those of the chemical reactions and the system will be under dynamic equilibrium.

For extremely rapid reactions, for example, radical reactions where there are short-lived radicals or photochemical reactions where excited-state molecules may play a significant role, the reactions may be diffusion-controlled, as their activation energies are <10 kJ mol^−1^, and the rate of the reaction depends on the dynamics of fast processes (proton transfer, solvation of the probe, solute, or counterion entry and exit from the aggregates) that take place in the nanoseconds to microsecond scales [37,55,56]. Some of these reactions, for instance, the bimolecular quenching of the excited state of the probes by other molecules present in the medium, have been used to determine the mobilities of molecules, to estimate local microviscosities and encounter probabilities in the medium, and to investigate ultrafast proton transfer reactions [37,56].

In summary, the rates of common thermally activated reactions are much lower than diffusion, and unless there are physical restrictions to movement (for example, if polymer networks are located in the interfacial region or if electrostatic forces are relevant), diffusion is not rate-limiting. Both diffusion-controlled and activation-controlled reactions are important and commonly observed in nature. Examples of diffusion-controlled reactions include heterogeneous catalyzes, cell metabolism, gas diffusion through solid, polymer chain growth kinetics, colloid or crystal growth, precipitation, fluorescence quenching and other photochemical processes, some electrochemical reactions, combustion, etc. In general, diffusion-controlled reactions are those with low activation energies, so that the reaction time is negligible in comparison with that required for the diffusion of the molecules to collide. On the contrary, if the reaction times are much higher than those required for the diffusion of the reactants (high activation energies), the reactions are controlled by activation.

Our research on antioxidants in fluid emulsions over the past decade demonstrates that the dynamic equilibrium condition holds provided that the emulsions are either kinetically stable or gently stirred after initial mixing because the intermolecular interactions between molecules and ions in emulsions are generally weak and their diffusivities should be orders of magnitude faster than the rates of thermal reaction [3,4,19,24,25,34,57,58,59,60,61,62,63].

### 3.2. Modeling Chemical Reactivity at the Interfaces of Emulsions

In bulk solution, reactants need to get close to each other to form the so-called “encounter complex” before the chemical reaction takes place, so that the observed (or measured) rate of a bimolecular reaction depends first on the relative rates of these processes. According to the transition state theory (TST), a bimolecular reaction A + B → Products is initiated when the formation of the “encounter pair” is followed by the formation of the activated complex, which must move on to the products for the reaction to proceed, otherwise the reaction does not take place [50,53]. The rate law is given by Equation (5), where the subscript T stands for total or stoichiometric concentration, and square brackets indicate moles per liter of solution volume. When the second reactant is in large excess, [B_T_] >> [A_T_], the observed rate is expressed by a first-order rate constant, *k*_obs_ (s^−1^) = *k*_2_[B_T_], where *k*_2_ is the second-order rate constant (M^−1^ s^−1^).

(5) $$r=-\frac{{d[A}_{T}]}{\mathrm{dt}}=k_{2}{[A}_{T}{][B}_{T}]=k_{\mathrm{obs}}{[A}_{T}]$$

In multiphasic systems, reactants (and other molecules) are exchanged between the different regions, Scheme 4B, and after bulk mixing is complete, the reactants distribute according to their relative solubilities between the oil, interfacial, and aqueous regions [19,24]. Assuming that the dynamic equilibrium conditions holds, the overall rate of the reaction is given by Equation (6).

(6) $$r=r_{W}+r_{I}+r_{O}=k_{W}(A_{W})(B_{W})\Phi_{W}+k_{I}(A_{I})(B_{I})\Phi_{I}+k_{O}(A_{O})(B_{O})\Phi_{O}$$

where subscripts W, I, and O indicate the oil, interfacial, and aqueous regions, respectively, *k*_W_, *k*_I_, and *k*_O_ are the second-order rate constants, parenthesis ( ) denote concentrations in moles per liter of the volume of a particular region, and Φ is the volume fraction of a region, defined as Φ = V_region_/V_Total_.

To correctly interpret chemical reactivity in colloidal systems, theoretical frameworks provided by different kinetic models are needed. Quantitative interpretations of experimental results, in terms of reactivity, can only be achieved if the local reagent concentrations and the intrinsic rate constants in the various regions can be obtained from the overall, apparent, experimental kinetic data. For instance, Menger and Portnoy [64] first developed pseudophase models to interpret chemical reactivity in micellar systems. The model was later expanded by Romsted [65,66,67] to incorporate ion-exchange effects. García-Río et al. developed kinetic models to quantitatively explain the influence of microemulsions on chemical reactivity [68] and Sanchez et al. employed the Bronsted relationship to rationalize chemical reactivity in microemulsions [69]. Certainly, it is up to the imagination of researchers to design and employ different chemical probes to assess the distributions of reactants of interest.

However, unraveling reaction mechanisms such as that depicted in Scheme 4B, whose overall rate *k*_obs_ is given by Equation (6), is extremely complex, and very intricate kinetic equations are frequently obtained because of the huge amount of variables needed to fully describe the partitioning of reactants and the rate of the reaction. Moreover, the rate constants in each region are distinct because the solvent properties of each region are totally different (differences may be of several orders of magnitude). To overcome the problem, judicious simplifications are made by selecting suitable chemical reactions under appropriate conditions. Most frequently, simplifications are achieved through: (1) a careful choice of the chemical structure of the reactants, for example, by employing hydrophobic or hydrophilic reactants, may favor the chemical reaction in one region with respect to the other ones [29,67,68,70,71,72], (2) by using chemical probes with specific molecular or spectral characteristics; (3) by choosing carefully and appropriate experimental conditions; (4) by using ionic surfactants to promote the concentration of reactants or its electrostatic separation in the interfacial region [28,73,74].

For example, N-nitroso-N-methy-p-toluensulfonamide has a well-established denitrosation mechanism [75], which was exploited to get insights towards the polarity of the Stern layer in micelles [76,77], the role of alcohols in micellar systems [77], and into the solvent properties of microemulsions [27]. An intelligent choice of molecular structures of reactants undergoing nitrosation and denitrosation reactions has also been exploited by Garcia-Río et al. [26,27] and Iglesias et al. [78,79] to investigate the use of microemulsions as reaction media. Aliaga et al. employed a series of 4-alkanoyloxy-1,1,6,6-tetramethylpyperidineoxyl (TEMPO) radicals of different amphiphilic characters as probes to investigate antioxidant activities in micelles and emulsions [80,81,82].

**Scheme 5:**
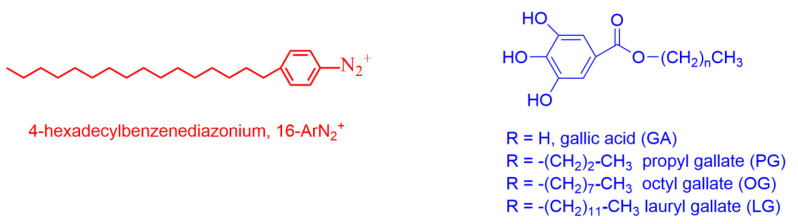
Structures of the chemical probe 16-ArN_2_^+^ and of some reactive antioxidants.

Romsted and coworkers [19,24,62,83] made use of the rich chemistry of arenediazonium ions to investigate the interfacial composition of association colloids and to investigate the distribution of polyphenolic antioxidants in intact emulsions under a variety of experimental conditions [19,24,62]. They synthesized the hydrophobic 4-hexadecylbenzenediazonium, 16-ArN_2_^+^, tetrafluoroborate, which is reduced in the presence of polyphenolic antioxidants to model the observed reactivity of the probe in emulsions and to estimate the distributions of the antioxidants, which otherwise could not be determined because of the (physical) impossibility of detaching the interfacial region from the aqueous and oil regions [24,84,85,86,87]. The chemical structures of 16-ArN_2_^+^ and of some representative antioxidants are shown in Scheme 5.

**Scheme 6:**
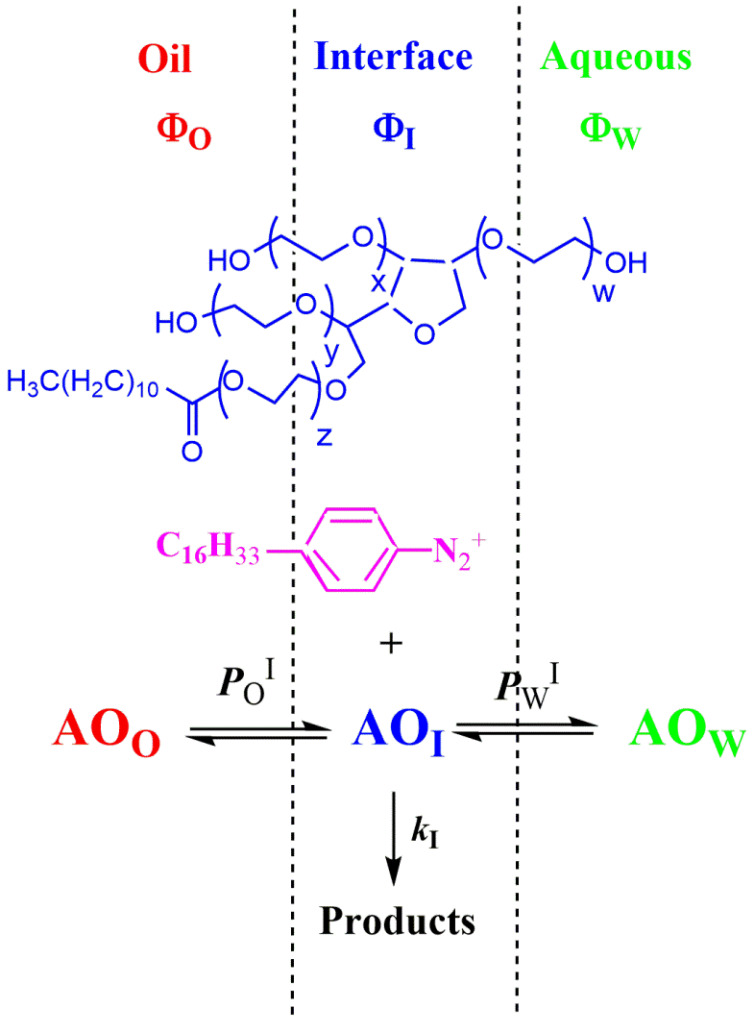
Dissection of a small portion of a non-ionic emulsion showing the oil (O), interfacial (I), and aqueous (W) regions. Because of the special molecular characteristics of the chemical probe, the reaction between 16-ArN_2_^+^ and the antioxidant takes place exclusively in the interfacial region of the emulsion. Φ indicates the volume fraction of a region, *P* is the partition constant, and *k*_I_ is the rate constant for the reaction between the chemical probe 16-ArN_2_^+^ and the antioxidant. The chemical structures of the surfactants Tween 20 and Span 80 are also shown.

The use of the 16-ArN_2_^+^ in emulsions considerably simplifies Scheme 4B. The compound 16-ArN_2_^+^ is both oil and water insoluble because it is a cation and because of its long hydrophobic tail. Therefore, it locates at the interfacial region of colloidal aggregates, so that their reactive group is anchored in the interfacial region of the emulsion where reacts with other molecules [62,83,88,89]. In this case, it is not necessary to consider the distribution of one of the reactants; the number of unknown parameters decreases significantly, and Scheme 6 now applies.

Because the reactive group of 16-ArN_2_^+^ is located only in the interfacial region, Scheme 6, and bearing in mind that the oil and water concentrations of 16-ArN_2_^+^ are negligible, i.e., [16-ArN_2_^+^_O_] and [16-ArN_2_^+^_W_] ≈ 0, Equation (6) simplifies to Equation (7). Kinetic runs are usually carried out under pseudo-first-order conditions, so that [AO_T_] >>> [16-ArN_2_^+^_T_]. In Equation (7), Φ_I_ is the surfactant volume fraction defined as Φ_I_ = V_surf_/V_total_.

(7) $$r=r_{I}=-\frac{d[{16-\mathrm{ArN}}_{2T}^{+}]}{dt}=k_{I}{(16-\mathrm{ArN}}_{2}^{+}{}_{T}{)(\mathrm{AO}}_{I}{)\Phi }_{I}$$

Scheme 6 can be further simplified by, for instance, carefully choosing the chemical characteristics of the antioxidant so that the antioxidant can be located exclusively in the interfacial region or can only partition between two regions. Some representative examples are given in the next section. For the sake of simplicity, we will first consider non-ionic emulsions, where the droplet surface is not charged and electrostatic effects are negligible, and will consider, in a different section, the case of ionic emulsions.

## 4. Main Equations Derived from the Application of the Pseudophase Model Emulsions

Predicting “a priori” the partitioning of a given antioxidant between the oil, interfacial, and aqueous regions of emulsions is truly challenging. Values of the partition constant in binary systems cannot be employed in emulsions because of the physical impossibility of isolating the interfacial region from the oil and aqueous ones, which makes it impossible to predict the values of the partition constants in emulsions ($P_{O}^{I}$ and $P_{W}^{I}$) from the value of the partition constant $P_{W}^{O}$ in binary systems. However, knowledge of $P_{W}^{O}$ values may, to a first approximation, help to envisage qualitatively how the distribution of the molecule may be in emulsions. Binary oil–water systems can be considered as limit cases of oil-in-water systems when no surfactant is added, that is, when the concentration of surfactant is low enough to allow phase separation. Two limiting situations can be envisaged, when the antioxidant is hydrophilic (low $P_{W}^{O}$ values), so that their concentration in the oil region is negligible, and the antioxidant only partitions between the aqueous and interfacial regions. Conversely, very hydrophobic antioxidants (high $P_{W}^{O}$ values) are water insoluble and only partition between the oil and interfacial regions. Cautions must be taken, however, because $P_{W}^{O}$ values for food-grade oils cannot be extrapolated from the values in octanol–water binary systems (probably the most common system employed to determine the hydrophobicity of a molecule) because the solvents are totally different [3,19,24,90].

### 4.1. Reactions in Non-Ionic Emulsions

#### 4.1.1. Reactions Where the Antioxidant Partitions between the Three Regions

Antioxidants of moderate hydrophobicity usually distribute between the three regions as shown in Scheme 6. Changes in the polarity of antioxidants can be achieved, for example, by grafting inert alkyl chains in the aromatic ring of polyphenols, so that homologous series of antioxidants bearing the same reactive groups but of different hydrophobicity can be prepared [91,92].

Combination of Equation (7) with the mass balance equations for AO and 16-ArN_2_^+^, and bearing in mind the definition of the relevant partition constants, Equations (8)–(10) can be derived for *k*_obs_ [24].

(8) $$P_{W}^{I}=\frac{{(\mathrm{AO}}_{I})}{{(\mathrm{AO}}_{W})}$$

(9) $$P_{O}^{I}=\frac{{(\mathrm{AO}}_{I})}{{(\mathrm{AO}}_{O})}$$

(10) $$k_{\mathrm{obs}}=\frac{\left[{\mathrm{AO}}_{T}\right]k_{I}P_{W}^{I}P_{O}^{I}}{\Phi_{O}P_{W}^{I}+\Phi_{{}_{I}}P_{W}^{I}P_{O}^{I}+\Phi_{W}P_{O}^{I}}$$

**Figure 4:**
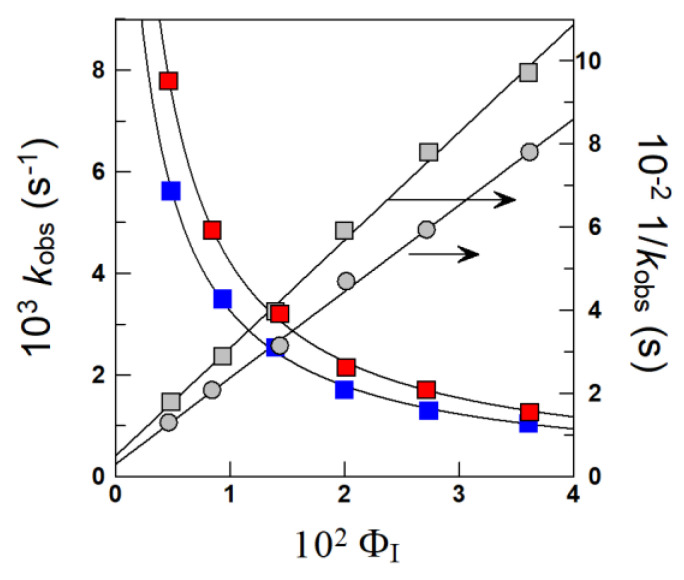
Variations of *k*_obs_ with Φ_I_ for methyl caffeate in 4:6 (squares) and 1:9 (circles) olive oil emulsions. The solid lines are the theoretical curves obtained by fitting the experimental data to Equations (16) and (17). Adapted from ref. [96].

Equation (10) encloses three unknowns, *k*_I_, $P_{O}^{I}$, and $P_{W}^{I}$, and their individual values cannot be obtained in a single experiment, and a second independent mathematical relationship is needed, which can be obtained by carrying out a second set of kinetic experiments in emulsions with a different Φ_W_/Φ_O_ ratio to obtain a second set of (*k*_obs_, Φ_I_) pairs of data. [25,93,94,95] After some mathematical treatment, Equation (11) can be derived, where the parameters ***a*** and ***b*** are defined by the Equations (12) and (13) [19,24,25]. The partition constants $P_{O}^{I}$ and $P_{W}^{I}$, defined by Equations (11) and (12), are then obtained by employing Equation (13) from two sets of kinetic data at different Φ_W_/Φ_O_ ratios and solving two equations in two unknowns. Once the partition constants are known, the *k*_I_ value is obtained from Equation (12). Figure 4 illustrates the variations of *k*_obs_ with Φ_I_ at two Φ_W_/Φ_O_ ratios.

(11) $$\frac{1}{k_{\mathrm{obs}}}=\frac{b}{a}\Phi_{I}+\frac{1}{a}$$

(12) $$a=\frac{{[\mathrm{AO}}_{T}]k_{I}P_{W}^{I}P_{O}^{I}{(1+\Phi }_{W}{/\Phi }_{O})}{P_{W}^{I}{+(\Phi }_{W}{/\Phi }_{O})P_{O}^{I}}$$

(13) $$b=\frac{P_{W}^{I}P_{O}^{I}{(1+\Phi }_{W}{/\Phi }_{O})}{P_{W}^{I}{+(\Phi }_{W}{/\Phi }_{O})P_{O}^{I}}$$

(14) $$\frac{P_{W}^{I}}{P_{O}^{I}}=\frac{{(\mathrm{AO}}_{O})}{{(\mathrm{AO}}_{W})}=P_{W}^{O}$$

A second, much more straightforward approach [25,97], is to measure the partition constant $P_{O}^{W}$ for the antioxidant distribution in binary oil–water mixtures because the value of this partition constant equals to the ratio of the partition constants $P_{W}^{I}$^/^$P_{O}^{I}$, Equation (15). This approach implies to consider the binary system as a constraining condition of the emulsion when the surfactant concentration goes to zero, i.e., when no surfactant is added, Scheme 2.

(15) $$\frac{P_{W}^{I}}{P_{O}^{I}}=\frac{{(\mathrm{AO}}_{O})}{{(\mathrm{AO}}_{W})}=P_{W}^{O}$$

Values for $P_{O}^{I}$ and $P_{W}^{I}$ are obtained by combining the value for $P_{O}^{W}$ (Equation (14)) with fits of *k*_obs_ versus Φ_I_ at constant [AO_T_] and Φ_W_:Φ_O_ (Equation (13)) and solving two equations in two unknowns, and once $P_{O}^{I}$ and $P_{W}^{I}$ are known, Equation (12) is used to determine *k*_I_. Further details on the calculations, advantages, and limitations of the approaches can be found elsewhere [25,93,94,95].

#### 4.1.2. Reactions Where the Antioxidant Partitions between Two Regions

**Scheme 7:**
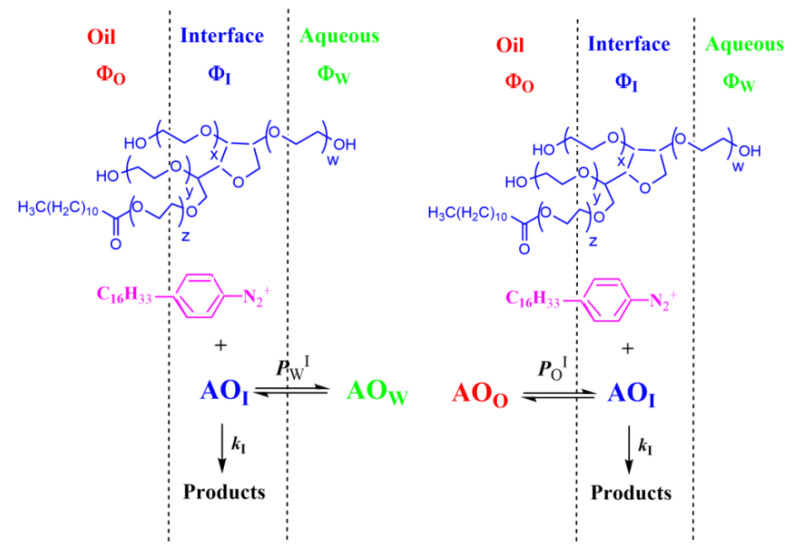
Distribution of a hydrophilic (oil insoluble) AO (**left**) and a hydrophobic (water-insoluble) antioxidant (**right**) in emulsions. $P_{W}^{I}$ and $P_{O}^{I}$ are the partition constants of the AO between the aqueous and interfacial and the oil and interfacial regions, respectively, and *k*_I_ is the interfacial rate constant for the reaction between 16-ArN_2_^+^ and AO.

We now consider those cases where the antioxidant only partitions between two regions. A number of polar antioxidants such as phenolic acids (gallic, caffeic, catechin) are oil insoluble and mainly distribute between the aqueous and interfacial region, Scheme 7. On the contrary, very hydrophobic antioxidants (such as α-tocopherol (vitamin E) and octyl and lauryl gallates) are water insoluble and only distribute between the oil and interfacial regions, Scheme 7. In both cases, only one partition constant is needed to describe their distribution: that between the water interface, $P_{W}^{I}$, and that between the oil and interfacial region, $P_{O}^{I}$, defined by Equations (8) and (9).

**Figure 5:**
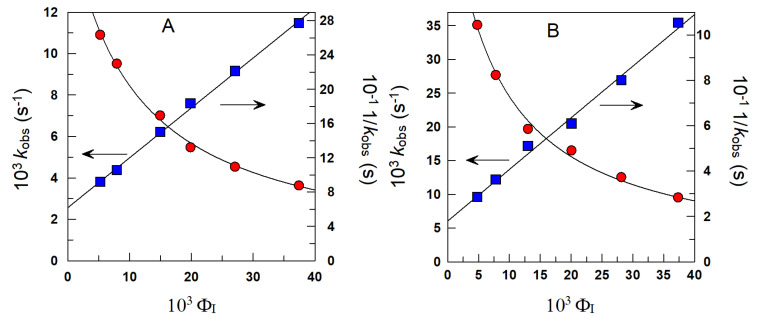
Illustrative examples of the variations of *k*_obs_ with Φ_I_ for the reaction between 16-ArN_2_^+^ with gallic acid (**A**) and with lauryl gallate (**B**) in 1:9 fish oil-in-water emulsions at T = 25 °C. The solid lines are the theoretical curves obtained by fitting the experimental data to Equations (16) and (18) (**A**) and (17) and (19) (**B**). Adapted from ref. [98].

Bearing in mind Equation (7), the definition of the partition constants, Equations (8) and (9), and the corresponding mass balances, Equations (16)–(19), can be derived. Equations (15) and (16) describe the dependence of *k*_obs_ on both the concentration of the antioxidant and the medium effects and were used to calculate $P_{W}^{I}$, *k*_I_ and $P_{O}^{I}$, *k*_I_ values, respectively. Equations (17) and (18) are the reciprocal of Equations (16) and (17), respectively, and predict that plots of 1/*k*_obs_ vs. Φ_I_ should be linear with a positive intercept. Figure 5 illustrates the variations of *k*_obs_ vs. Φ_I_ and 1/*k*_obs_ vs. Φ_I_ for gallic acid and lauryl gallate, and values for *k*_I_, $P_{W}^{I}$ and $P_{O}^{I}$ are obtained from the slopes and intercepts of Equations (18) and (19).

(16) $$k_{\mathrm{obs}}=\frac{k_{I}{\left[\mathrm{AO}\right]}_{T}P_{W}^{I}}{\Phi_{I}P_{W}^{I}+\Phi_{W}}$$

(17) $$k_{\mathrm{obs}}=\frac{k_{I}{\left[\mathrm{AO}\right]}_{T}P_{O}^{I}}{\Phi_{I}P_{O}^{I}{+\Phi }_{O}}$$

(18) $$\frac{1}{k_{\mathrm{obs}}}=\frac{\Phi_{W}}{k_{I}{\left[\mathrm{AO}\right]}_{T}P_{W}^{I}}+\frac{1}{k_{I}{\left[\mathrm{AO}\right]}_{T}}\Phi_{I}$$

(19) $$\frac{1}{k_{\mathrm{obs}}}=\frac{\Phi_{O}}{k_{I}{\left[\mathrm{AO}\right]}_{T}P_{O}^{I}}+\frac{1}{k_{I}{\left[\mathrm{AO}\right]}_{T}}\Phi_{I}$$

#### 4.1.3. Reactions Taking Place Exclusively at the Interfacial Region

**Scheme 8:**
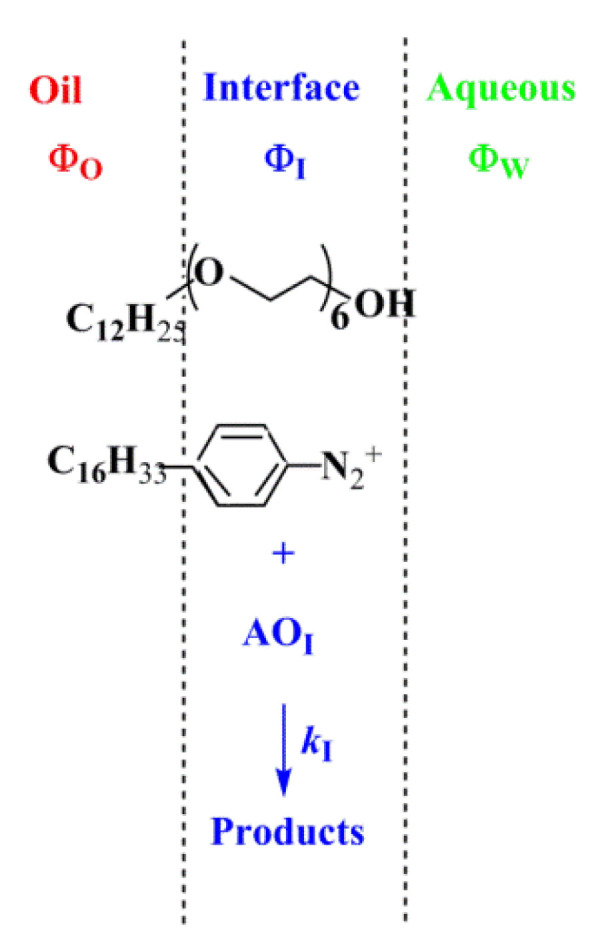
Limiting case where both reactants do not partition and are located in the interfacial region of a non-ionic emulsion.

This represents a limiting case in which both reactants (16-ArN_2_^+^ and the antioxidant) do not partition, as illustrated in Scheme 8, and therefore, are found entirely in the interfacial region.

**Figure 6:**
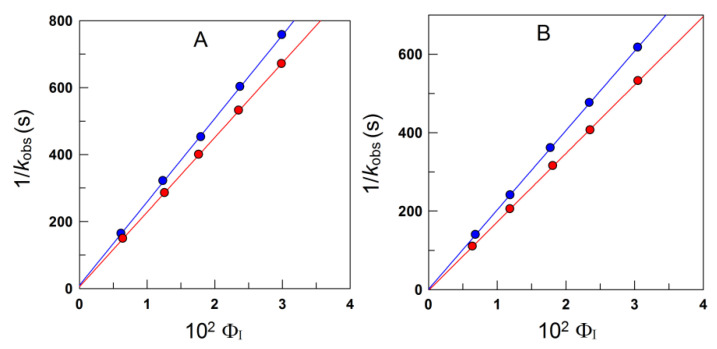
Variations of 1/*k*_obs_ with Φ_I_ in 1:9 (blue) and 1:4 (red) *v*:*v* octane-in-water emulsions for the reaction of 16-ArN_2_^+^ with octyl (**A**) and lauryl (**B**) gallate. Extracted from ref. [99].

This case is formally equivalent to that of a bimolecular reaction taking place in a vessel of volume equal to that of the interfacial region, and the observed rate constant for the reaction is given by Equation (20), which predicts that *k*_obs_ values should decrease upon increasing Φ_I_, as illustrated in Figure 6.

(20) $$k_{\mathrm{obs}}=k_{2}{[\mathrm{AO}}_{T}]=\frac{k_{I}{[\mathrm{AO}}_{T}]}{\Phi_{I}}$$

The reciprocal of Equation (20) predicts that plots of 1/*k*_obs_ vs. Φ_I_ must be straight lines with intercepts at 1/*k*_obs_ = 0 as illustrated in Figure 6. Some representative values of *k*_I_, obtained by least squares fitting of (1/*k*_obs_, Φ_I_) pairs of data, are shown in Table 3.

Because the values of *k*_I_ reflect the polarity of the reaction site, the small variations in *k*_I_ upon changing the oil/water ratio suggest they have negligible effects on the polarity of the interfacial region of both emulsions. The results in Table 3 also suggest that changes in the length of the alkyl chain of the antioxidant have negligible effects on *k*_I_ values.

### 4.2. Reactions in Ionic Emulsions

**Scheme 9:**
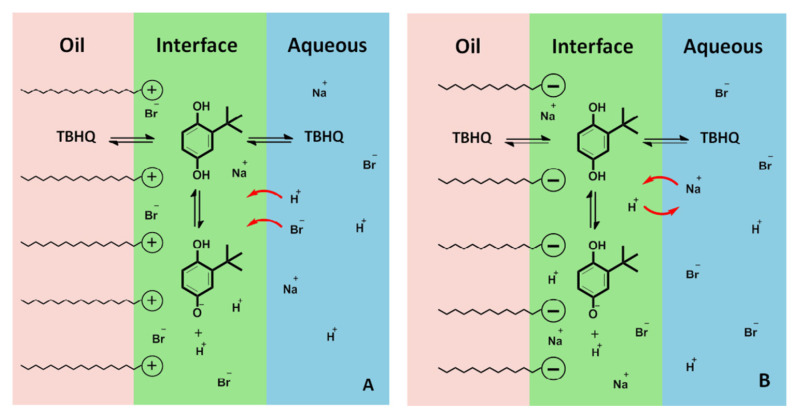
Outlines of the application of the pseudophase model to ionic emulsions illustrating: (**A**) the Donnan equilibrium in CTAB emulsions and (**B**) the ion exchange in SDS emulsions. TBHQ stands *t*-butylhydroquinone. Reproduced from ref. [87].

When the surfactants employed to stabilize kinetically emulsions are ionic, the surfaces droplets become either negatively or positively charged, Scheme 9, and this charge has significant kinetic consequences because:

(1) Electrostatic forces become important because droplet interfaces may attract or repel ions present in the aqueous phase, decreasing their concentration substantially or producing high local concentrations [30,83].

(2) Charged interfaces significantly modify the interfacial molarities of ions such as H^+^ or OH^−^, having major effects on the p*K*_a_ of weak acids present in emulsions. Anionic interfaces have higher interfacial H^+^ concentrations relative to those in the bulk, but decrease the interfacial OH^−^ concentration with respect to that in the bulk aqueous phase. Cationic emulsions do the opposite [30,65,100,101].

The pseudophase ion exchange (PIE) model provides a suitable theoretical and operative framework to interpret chemical reactivity at charged emulsion interfaces. The PIE model was first developed for modeling chemical reactivity in micellar solutions of ionic surfactants (Scheme 2) to describe specific ion effects and ion distributions [65,67,100]. The PIE model assumes that the interfacial regions of ionic colloidal systems behave as selective ion exchangers, where counterions (ions of opposite charge to that of the surface) exchange with H^+^. For example, in anionic sodium dodecyl sulfate micelles, Na^+^ ions are counterions, as well as those from added NaCl. The dynamic equilibria of counterions between the aqueous and interfacial regions, Equation (21), is defined by an empirical ion exchange constant *K*_H_^Na+^, as in ion exchange resins, Equation (22) [67,102,103,104].

(21) $$H_{I}^{+}{+\mathrm{Na}}_{W}^{+}\overset{K_{H}^{\mathrm{Na}}}{\;⇄}{\;H}_{W}^{+}{+\mathrm{Na}}_{I}^{+}$$

(22) $$K_{H}^{\mathrm{Na}}=\frac{{[H}_{W}^{+}{][\mathrm{Na}}_{I}^{+}]}{{[H}_{I}^{+}{][\mathrm{Na}}_{W}^{+}]}$$

(23) $$H_{I}^{+}{+\mathrm{Br}}_{I}^{-}\overset{K_{H}^{\mathrm{Br}}}{\;⇄}{\;H}_{W}^{+}{+\mathrm{Br}}_{W}^{-}$$

(24) $$K_{H}^{\mathrm{Br}}=\frac{{[H}_{W}^{+}{][\mathrm{Br}}_{W}^{-}]}{{[H}_{I}^{+}{][\mathrm{Br}}_{I}^{-}]}$$

In acidic solutions of cationic micelles, the H^+^ is a co-ion and a Donnan equilibrium such as that depicted in Equation (23) is used to describe the effect of added Br^−^ on the proton distribution [105]. The Donnan equilibrium constant, $K_{H}^{\mathrm{Na}}$, Equation (24), is employed to define the effects of added counterions, Br^−^, on the H^+^ co-ion distribution between the interfacial and aqueous regions. Equation (24) states that, upon addition of NaBr, the Br^−^ concentration in the aqueous region increases, increasing the Br^−^ and H^+^ concentration in the interfacial region. However, the ratio of the numerator and denominator, Equation (24), remains constant [65,67,103].

**Figure 7:**
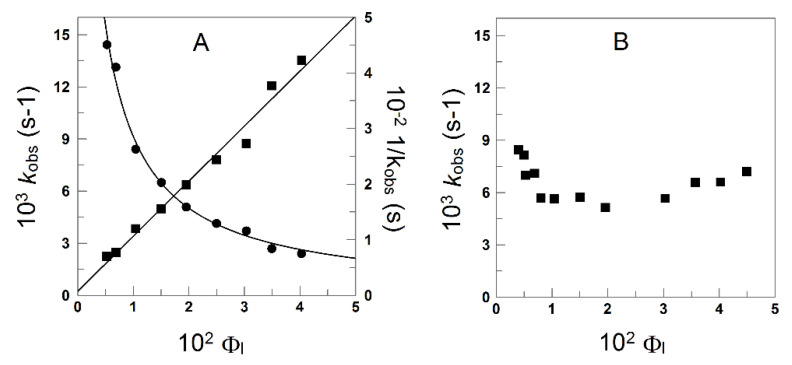
Changes in *k*_obs_ upon increasing Φ_I_ for the reaction between 16-ArN_2_^+^ and TBHQ in (**A**) CTAB emulsions and (**B**) in SDS emulsions. Figure extracted from ref. [87].

Figure 7 illustrates the kinetic profiles obtained for the reaction between the antioxidant *t*-butylhydroquinone and 16-ArN_2_^+^ in ionic cetyltrimethylammonium (CTAB) and sodium dodecyl sulfate, SDS, emulsions [85,87]. In CTAB emulsions, Figure 7A, in the absence of salt, the decrease in *k*_obs_ with increasing Φ_I_ is produced by dilution of TBHQ upon increasing the interfacial volume (i.e., increasing surfactant concentration) [19].

The observed effects on *k*_obs_ depend on the nature of the reaction considered. For the reaction between ArN_2_^+^ ions with antioxidants, ion exchange has important consequences because most antioxidants are (poly)phenolic compounds and the reactive species is the deprotonated form of the phenol, AO^−^ [106]. Thus, at constant [AO_T_] and Φ_O_:Φ_W_, the main effect of increasing Φ_I_ (i.e., addition of CTAB) on *k*_obs_ is to dilute both the anionic and neutral forms of TBHQ. The effective H^+^ concentration in the interfacial region (mole per liter of interfacial volume) should be reduced to approximately the same amount as the TBHQ and TBHQ^−^ concentrations. Therefore, the decrease in *k*_obs_ with increasing Φ_I_ is caused by dilution of the TBHQ anion within the increasing volume of the totality of the interfacial regions of the emulsions droplets as in nonionic emulsions, Figure 7A [3,19,85].

In SDS emulsions, Figure 7B, the variation of *k*_obs_ with Φ_I_ decreases initially, passing through a shallow minimum, and then increases. The shallow minimum was attributed to two competing effects, the simultaneous decrease in H^+^ concentration in the interfacial region and the dilution of TBHQ^−^, which leads to an increase in *k*_obs_ because of the increase in the concentration of the reactive species TBHQ^−^. Details can be found elsewhere [19,85,87].

## 5. Effects of Temperature on the Kinetics in Emulsions: Interfacial Activation Parameters

The activation parameters for the reaction of 16-ArN_2_^+^ with antioxidants in the interfacial region of emulsions can be obtained from the variations of the rate constant *k*_I_ (see Scheme 6) at different temperatures [107], which are obtained as described in Section 4.1. This is the only method reported in the literature, up to date, that permits estimations of activation energies of reactions taking place in the interfacial region of emulsified systems [59,63,108,109].

**Figure 8:**
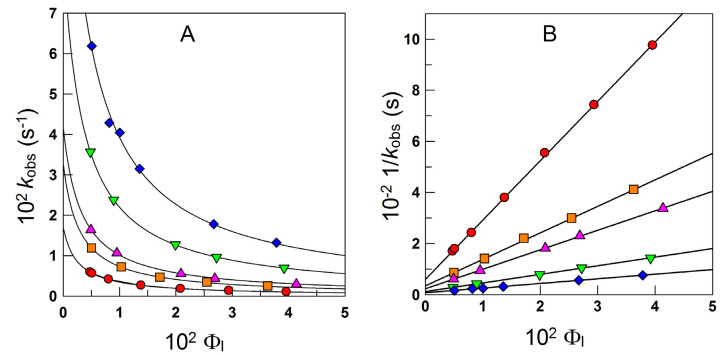
Effects of increasing temperature (°C) on the reaction of caffeic acid with 16-ArN_2_^+^ in 1:9 (O:W) corn oil-in-water emulsions stabilized with Tween 20. The solid lines were obtained by fitting the variations of *k*_obs_ (**A**) and 1/*k*_obs_ (**B**) with Φ_I_ to Equations (12)–(15). Reprinted from ref. [63].

Figure 8A illustrates the effect of temperature on *k*_obs_ for the reaction of 16-ArN_2_^+^ with caffeic acid at increasing Φ_I_ values. The straight lines shown in Figure 8B are plots of 1/*k*_obs_ vs. Φ_I_, from where the *k*_I_ values were obtained (Equations (12)–(15)). Similar plots were obtained when employing different antioxidants, [59,63,108,109], and Table 4 shows some values for interfacial rate constants *k*_I_ for α-tocopherol, caffeic acid and propyl (PG), octyl (OG) and lauryl (LG) gallates (see Scheme 5) at different temperatures.

**Figure 9:**
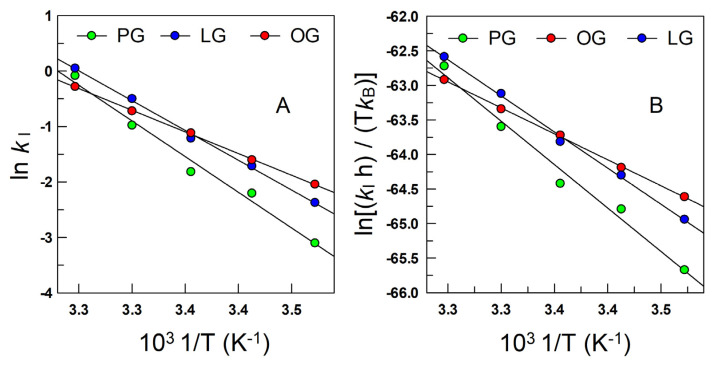
Variations in ln *k*_I_ and ln(*hk*_I_/*k*_B_T) as a function of 1/T (Kelvin) for the reaction of propyl (PG), octyl (OG), and lauryl (LG) gallates with 16-ArN_2_^+^ in the interfacial region of 1:9 (O/W) corn oil emulsions. Figure extracted from ref. [59].

The activation energy, *E*_a_, and the activation parameters, Δ*H*^‡^ and Δ*S*^‡^, for the reaction in the interfacial region were determined by employing the Arrhenius Equation (25) and that derived from the absolute rate theory, Equation (26), where *k*_B_ and *h* refers to the Boltzmann and Plank constants, respectively. Illustrative examples are given in Figure 9.

(25) $$\ln k_{I}=A-\frac{E_{a}}{RT}$$

(26) $$\ln\frac{hk_{I}}{k_{B}T}=\frac{\Delta S^{\mp }}{R}-\frac{\Delta H^{\mp }}{RT}$$

## 6. Conclusions and Perspective

Understanding the reactivity and behavior of solute molecules at the interface between immiscible liquids (oil and water) is very important at both the fundamental and theoretical levels because the anisotropic environment of the interfacial region is expected to affect the course of chemical reactions in a way significantly different from that in bulk fluids. Significant differences are also expected from those reactions taking place in a single bulk adjacent to a reactive interface (such as in heterogeneous catalysis). The latter requires the arrival of only one molecule at the interface; meanwhile, reactions taking place in three-dimensional interfaces such as those in emulsions require the formation of an encounter pair, i.e., need the simultaneous arrival at the same location within the interface of two reactive species.

By combining a careful choice of reactants, their properties, and experimental conditions with the theoretical framework provided by pseudophase models, new approaches to model chemical reactivity in three-dimensional interfaces can be obtained. Pseudophase models are powerful tools to model chemical reactivity and to correctly interpret experimental kinetic data, provided that the specificity of the reaction systems is incorporated into the model [71,110,111]. They can be applied to a variety of reactive systems and to reactions carried out under restricted geometry conditions. In fact, the basic equations derived from the application of the pseudophase model are, formally, similar to the mathematical relationships obtained for enzymatic reactions [112,113], and those to model heterogeneous catalysis [50]. However, the use of the pseudophase models requires considering the effects of surface potentials and partitioning effects between regions.

Unfortunately, no complete and systematic theory exists for modeling surface potentials nor solvation effects, and both theoretical and experimental studies of chemical reactions taking place at liquid interfaces are still far from being completely understood, and more experimental data and theoretical models are needed to provide a full understanding of the reaction dynamics at interfaces [114,115,116]. On the theoretical side, progress is needed to understand how the structure of the neat water/organic interface is composed, and how it influences reactant and/or ion transfers and their solvation. The molecular orientation of the reactants at the interface is also relevant because the interfacial region is anisotropic, and certain molecular orientations may be preferred at the interface. A deeper understanding of solvation at liquid interfaces is also important for the correct interpretation of the interfacial phenomena. Solvation plays a central role in understanding interfacial effects on chemical reactions, because it depends strongly on the solvation free energy of the reactants, products, and of the transition state [114].

## Figures and Tables

**Table 1:** Applications and corresponding HLB values. Adapter from refs. [32,33].

HLB Range	Application
<3	Surface films
3–6	Water-in-oil emulsions
7–9	Wetting agents
8–15	Oil-in-water emulsions
13–15	Detergents
15–18	Solubilizers

**Table 2:** Typical values of the rate constants obtained for bimolecular reactions in aqueous solution at *T* = 25 °C as a function of their *E*_a_ values. Data from Van Boeckel [51,52].

*E*_a_ (kJ mol^−1^)	*k*_chem_ (dm^3^ mol^−1^ s^−1^)
~0	~10^10^
<25	10^9^–10^10^
50	10^5^
100	10^−4^
>200	<10^−22^

**Table 3:** Some representative values of the interfacial rate constant for the reaction between 16-ArN_2_^+^ and octyl and lauryl gallates (OG and LG, respectively) in octane-based emulsions. Note that the intercept is statistically zero as predicted by Equation (9). Data extracted from ref. [99].

AO	Emulsion	Intercept	10^2^ *k*_I_ (M^−1^ s^−1^)
OG	1:1	5 ± 5	1.0 ± 0.1
OG	1:4	2 ± 2	1.1 ± 0.2
LG	1:1	1 ± 1	1.2 ± 0.1
LG	1:4	−2 ± 2	1.4 ± 0.3

**Table 4:** Values for the interfacial rate constant *k*_I_ obtained at different temperatures for the reaction of 16-ArN_2_^+^ with caffeic acid (CA), α-tocopherol (TOC), propyl (PG), octyl (OG), and lauryl (LG) gallates. Data extracted from refs. [59,63].

	CA	TOC	PG	OG	LG
T (°C)	10^2^ *k*_I_ (M^−1^ s^−1^)	10^2^ *k*_I_ (M^−1^ s^−1^)	10^2^ *k*_I_ (M^−1^ s^−1^)	10^2^ *k*_I_ (M^−1^ s^−1^)	10^2^ *k*_I_ (M^−1^ s^−1^)
15	0.97 ± 0.01	---	4.5 ± 0.1	12.9 ± 0.1	9.3 ± 0.2
20	2.15 ± 0.04	9.2 ± 0.5	11 ±0.1	20 ± 1	18 ± 1
25	3.03 ± 0.02	16.8 ± 0.5	16 ± 0.5	33 ± 1	29 ± 1
30	6.64 ± 0.03	21 ± 0.4	37 ± 4	48 ± 3	61 ± 4
35	12.1 ± 0.05	37.6 ±0.6	91 ± 2	75 ± 5	105 ± 10

## Data Availability

Not applicable.

## References

[1] Shahidi F. (2015). Handbook of Antioxidants for Food Preservation.

[2] Frankel E.N. (2005). Lipid Oxidation.

[3] Costa M., Losada-Barreiro S., Paiva-Martins F., Bravo-Díaz C. (2021). Polyphenolic Antioxidants in Lipid Emulsions: Partitioning Effects and Interfacial Phenomena. Foods.

[4] Losada-Barreiro S., Bravo-Díaz C., Paiva-Martins F., Aboudzadeh M.A. (2021). Why encapsulate antioxidants in emulsion-based systems, where they are located, and how location affects their efficiency. Emulsion-Based Encapsulation of Antioxidants.

[5] Larsson K., Fribera S.E. (1990). Food Emulsions.

[6] Raman M., Almutairdi A., Mulesa L., Alberda C., Beattie C., Gramlich L. (2017). Parenteral Nutrition and Lipids. Nutrients.

[7] Sala-Vila A., Barbosa V.M., Calder P.C. (2007). Olive oil in parenteral nutrition. Curr. Opin. Clin. Nutr. Metab. Care.

[8] Gupta A., Eral H.B., Hatton T.A., Doyle P.S. (2016). Nanoemulsions: Formation, properties and applications. Soft Matter.

[9] McClements D.J., Rao J. (2011). Food-Grade Nanoemulsions: Formulation, Fabrication, Properties, Performance, Biological Fate, and Potential Toxicity. Crit. Rev. Food Sci. Nutr..

[10] McClements D.J. (2010). Emulsion Design to Improve the Delivery of Functional Lipophilic Components. Annu. Rev. Food Sci. Technol..

[11] Grumezescu A.M., Grumezescu A.M. (2016). Emulsions, Nanotechnology in the Agri-Food Industry.

[12] Friberg S.E., Larsson K. (1997). Food Emulsions.

[13] McClements D.J. (2015). Food Emulsions: Principles, Practices and Techniques.

[14] Coupland J.N., McClements D.J. (1996). Lipid oxidation in food emulsions. Trends Food Sci. Technol..

[15] Laguerre M., Bily A., Birtić S., Galanakis C.M. (2020). Lipid oxidation in food. Lipids and Edible Oils.

[16] Berton-Carabin C.C., Ropers M.-H., Genot C. (2014). Lipid Oxidation in Oil-in-Water Emulsions: Involvement of the Interfacial Layer. Compr. Rev. Food Sci. Food Saf..

[17] McClemments D.J., Decker E.A. (2000). Lipid Oxidation in Oil-in Water Emulsions: Impact of Molecular Environment on Chemical Reactions in Heterogeneous Food Systems. J. Food Sci..

[18] Berton C., Ropers M.H., Viau M., Genot C. (2011). Contribution of the interfacial layer to the protection of emulsified lipids against oxidation. J. Agric. Food Chem..

[19] Bravo-Díaz C., Romsted L.S., Liu C., Losada-Barreiro S., Pastoriza-Gallego M.J., Gao X., Gu Q., Krishnan G., Sánchez-Paz V., Zhang Y. (2015). To Model Chemical Reactivity in Heterogeneous Emulsions, Think Homogeneous Microemulsions. Langmuir.

[20] Weiss J. (2002). Unit D.35: Key Concepts of Interfacial Properties in Food Chemistry. Current Protocols in Food Analytical Chemistry, Supplement.

[21] Calder P.C., Waitzberg D.L., Klek S., Martindale R.G. (2020). Lipids in Parenteral Nutrition: Biological Aspects. J. Parenter. Enter. Nutr..

[22] de Melo Barbosa R., Severino P., Finkler C.L.L., de Paula E., Holban A.-M., Grumezescu A.M. (2019). Lipid-based colloidal carriers for transdermal administration of bioactives. Materials for Biomedical Engineering.

[23] Ghosh N., Das A., Chaffee S., Roy S., Sen C.K., Chatterjee S., Jungraithmayr W., Bagchi D. (2018). Reactive Oxygen Species, Oxidative Damage and Cell Death. Immunity and Inflammation in Health and Disease.

[24] Romsted L.S., Bravo-Díaz C. (2013). Modelling chemical reactivity in emulsions. Curr. Opin. Colloid Interface Sci..

[25] Gunaseelan K., Romsted L.S., Pastoriza-Gallego M.J., González-Romero E., Bravo-Díaz C. (2006). Determining a-tocopherol distributions betweeen the Oil, Water and Interfacial Regions of Macroemulsions: Novel Applications of Electrocanalytical Chemistry and a Pseudophase Kinetic Model. Adv. Colloid Interface Sci..

[26] Astray G., Cid A., Garcia Rio L., Hervella P., Mejuto J.C., Pérez-Lorenzo M. (2008). Organic reactivity in AOT-stabilized microemulsions. Prog. React. Kin. Mech..

[27] Lopez-Quintela M.A., Tojo C., Blanco M.C., Garcia Rio L., Leis J.R. (2004). Microemulsion dynamics and reactions in microemulsions. Curr. Opin. Colloid Interface Sci..

[28] Garti N. (2003). Microemulsions as microreactors for food applications. Curr. Opin. Colloid Interface Sci..

[29] Bunton C., Yao J., Romsted L.S. (1997). Micellar Catalysis: A useful misnomer. Curr. Opin. Colloid Interface Sci..

[30] Khan M.N., Science S. (2007). Micellar Catalysis.

[31] Laguerre M., Bily A., Roller M., Birtić S. (2017). Mass Transport Phenomena in Lipid Oxidation and Antioxidation. Annu. Rev. Food Sci. Technol..

[32] McClements D.J. (2005). Food Emulsions.

[33] Tadros T.F. (1984). Surfactants.

[34] Costa M., Freiría-Gándara J., Losada-Barreiro S., Paiva-Martins F., Bravo-Díaz C. (2020). Effects of droplet size on the interfacial concentrations of antioxidants in fish and olive oil-in-water emulsions and nanoemulsions and on their oxidative stability. J. Colloid Interface Sci..

[35] McClements D.J. (2018). Enhanced delivery of lipophilic bioactives using emulsions: A review of major factors affecting vitamin, nutraceutical and lipid bioaccessibility. Food Funct..

[36] García-Pérez P., Lozano-Milo E., Gallego P.P., Tojo C., Losada-Barreiro S., Bravo-Díaz C., Milani J.M. (2018). Plant Antioxidants in Food Emulsions. Some New Aspects of Colloidal Systems in Foods.

[37] Quina F.H. (2013). Dynamics and prototropic reactivity of electronically excited states in simple surfactant aggregates. Curr. Opin. Colloid Interface Sci..

[38] Malassagne-Bulgarelli N., McGrath K.M. (2013). Emulsion ageing: Effect on the dynamics of oil exchange in oil-in-water emulsions. Soft Matter.

[39] Skhiri Y., Gruner P., Semin B., Brosseau Q., Pekin D., Mazutis L., Goust V., Kleinschmidt F., El Harrak A., Hutchison J.B. (2012). Dynamics of molecular transport by surfactants in emulsions. Soft Matter.

[40] Malassagne-Bulgarelli N., McGrath K.M. (2009). Dynamics of oil transfer in oil-in-water emulsions. Soft Matter.

[41] Wooster T.J., Golding M., Sanguansri P. (2008). Impact of Oil Type on Nanoemulsion Formation and Ostwald Ripening Stability. Langmuir.

[42] Colafemmina G., Palazzo G., Ceglie A., Ambrosone L., Cinelli G., Di Lorenzo V. (2002). Restricted Diffusion: An Effective Tool to Investigate Food Emulsions. Prog. Colloid Polym. Sci..

[43] Soderman O., Nyden M. (1999). NMR translational diffusion studies of a model microemulsion. Colloid Surf. A: Physicochem. Eng. Aspects.

[44] Levinger N.E. (2000). Ultrafast dynamics in reverse micelles, microemulsions, and vesicles. Curr. Opin. Colloid Interface Sci..

[45] Kabalnov A.S. (1994). Can Micelles Mediate a Mass Transfer between Oil Droplets?. Langmuir.

[46] Smoluchowski M.V. (1918). Versuch Einer Mathematischen Theorie der Koagulationskinetik kolloider Losungen. Z. Phys. Chem..

[47] Andraos J. (2008). The Contributions of Solomon F. Acree (1875–1957) and the Centennial Anniversary of the Discovery of the Acree–Curtin–Hammett Principle. Chem. Educator.

[48] Bravo-Díaz C., Romsted L.S., Losada-Barreiro S., Paiva-Martins F. (2017). Using a pseudophase model to determine AO distributions in emulsions: Why dynamic equilibrium matters. Eur. J. Lipid Sci. Technol..

[49] Anslyn E.V., Dougherty D.A. (2006). Modern Physical Organic Chemistry.

[50] Laidler K.J. (1987). Chemical Kinetics.

[51] Van Boekel M.A.J.S. (2009). Kinetic Modeling of Reactions in Foods.

[52] van Boekel M.A.J.S. (2008). Kinetic Modeling of Food Quality: A Critical Review. Compr. Rev. Food Sci. Food Saf..

[53] Espenson J.H. (1995). Chemical Kinetics and Reaction Mechanisms.

[54] Atkins P., de Paula J. (2010). Physical Chemistry.

[55] Pace T.C.S., Bohne C., Richard J.P. (2007). Dynamics of guest binding to supramolecular systems: Techniques and selected examples. Advances in Physical Organic Chemistry.

[56] Quina F.H., Lissi E.A. (2004). Photoprocesses in Microaggregates. Acc. Chem. Res..

[57] Meireles M., Losada-Barreiro S., Costa M., Paiva-Martins F., Bravo-Díaz C., Monteiro L.S. (2020). Control of antioxidant efficiency of chlorogenates in emulsions: Modulation of antioxidant interfacial concentrations. J. Sci. Food Agric..

[58] Costa M., Losada-Barreiro S., Bravo-Díaz C., Vicente A.A., Monteiro L.S., Paiva-Martins F. (2020). Influence of AO chain length, droplet size and oil to water ratio on the distribution and on the activity of gallates in fish oil-in-water emulsified systems: Emulsion and nanoemulsion comparison. Food Chem..

[59] Raimúndez-Rodríguez E.A., Losada-Barreiro S., Bravo-Díaz C. (2019). Enhancing the fraction of antioxidants at the interfaces of oil-in-water emulsions: A kinetic and thermodynamic analysis of their partitioning. J. Colloid Interface Sci..

[60] Mitrus O., Żuraw M., Losada-Barreiro S., Bravo-Díaz C., Paiva-Martins F. (2019). Targeting Antioxidants to Interfaces: Control of the Oxidative Stability of Lipid-Based Emulsions. J. Agric. Food Chem..

[61] Losada-Barreiro S., Bravo-Díaz C. (2017). Free radicals and polyphenols: The redox chemistry of neurodegenerative diseases. Eur. J. Med. Chem.

[62] Dar A.A., Bravo-Diaz C., Nazir N., Romsted L.S. (2017). Chemical kinetic and chemical trapping methods: Unique approaches for determining respectively the antioxidant distributions and interfacial molarities of water, counter-anions, and other weakly basic nucleophiles in association colloids. Curr. Opin. Colloid Interface Sci..

[63] Losada-Barreiro S., Sánchez-Paz V., Bravo-Díaz C. (2015). Transfer of antioxidants at the interfaces of model food emulsions: Distributions and thermodynamic parameters. Org. Biomol. Chem..

[64] Menger F.M., Portnoy C.E. (1967). Chemistry of reactions proceeding inside molecular aggregates. J. Am. Chem. Soc..

[65] Romsted L.S., Mittal K.L., Lindman J. (1984). Micellar Effects on Reaction Rates and Equilibria. Surfactants in Solution.

[66] Romsted L.S., Zanette D. (1988). Quantitative Treatment of Indicator Equilibria in Micellar Solutions of Sodium Decyl Phosphate amd Sodium Dodecyl Sulfate. J. Phys. Chem..

[67] Bunton C.A., Nome F., Quina F.H., Romsted L.S. (1991). Ion Binding and Reactivity at Charged Aqueous Interfaces. Acc. Chem. Res..

[68] Garcia-Rio L., Leis J.R., Mejuto J.C., Perez-Lorenzo M. (2007). Microemulsions as microreactors in physical organic chemistry. Pure Appl. Chem..

[69] Muriel-Delgado F., Jiménez R., Gómez-Herrera C., Sánchez F. (1999). Use of the Brönsted Equation in the Interpretation of Micellar Effects in Kinetics (II). Study of the Reaction [Fe(CN)_5_(4-CNpy)]^3−^ + CN^−^ ⇄ Fe(CN)_6_^4−^ + 4-CNpy in CTACl Micellar Solutions. Langmuir.

[70] Blasko A., Bunton C.A., Hong Y.S., Mhala M.M., Moffat J.R., Wright S. (1991). Micellar Rate Effects on Reactions of Hydroxide Ion with Phosphinate and Thiophosphinate Esters. J. Phys. Org. Chem..

[71] Bunton C.A. (2006). The dependence of micellar rate effects upon reaction mechanism. Adv. Colloid Interface Sci..

[72] Bunton C.A., Hong Y.-S., Romsted L.S., Mittal K.L., Fendler E.J. (1982). A Quantitative Treatment of the Deprotonation Equilibria of Benzimidazole in Basic Solutions of Cetyltrimethylammonium Ion (CTAX) Surfactants. Solution Behaviour of Surfactants, Theoretical and Applied Aspects.

[73] Behera G., Mishra B., Behera P., Panda M. (1999). Fluorescent probes for structural and distance effect studies in micelles, reversed micelles and microemulsions. Adv. Colloid Interface Sci..

[74] Shoemaker R., Holmberg K., Stubenrauch S. (2009). Reactions in organized surfactant systems. Microemulsions: Background, New Concepts, Applications, Perspectives.

[75] Williams D., Lyn H. (1976). Denitrosation of N-Methyl-N-nitrosotoluene-p-sulphonamide in Acid Solution. J. Chem. Soc. Perkin Trans. II.

[76] Bravo C., Herves P., Leis J.R., Peña M.E. (1990). Micellar Effects in the Acid Denitrosation of N-Nitroso-N-methyl-p-toluensulfonamide. J. Phys. Chem..

[77] Bravo C., Leis J.R., Peña M.E. (1992). Effect of Alcohols on Catalysis by Dodecyl Sulfate Micelles. J. Phys. Chem..

[78] Iglesias E., Garcia-Rio L., Leis J.R., Casado J. (1997). Nitrosation reactions and nitrosocompounds in microheterogeneous media. Recent Res. Devel. Phys. Chem..

[79] Iglesias E. (1998). Cyclodextrins as Enzyme Models in Nitrosation and in Acid−Base-Catalyzed Reactions of Alkyl Nitrites. J. Am. Chem. Soc..

[80] Aliaga C., Bravo-Moraga F., Gonzalez-Nilo D., Márquez S., Lürh S., Mena G., Rezende M.C. (2016). Location of TEMPO derivatives in micelles: Subtle effect of the probe orientation. Food Chem..

[81] Aliaga C., Celis F., Lürh S., Oñate R. (2015). TEMPO-Attached Pre-fluorescent Probes Based on Pyridinium Fluorophores. J. Fluoresc..

[82] Lopez de Arbina A., Losada-Barreiro S., Rezende M.C., Vidal M., Aliaga C. (2019). The location of amphiphobic antioxidants in micellar systems: The diving-swan analogy. Food Chem..

[83] Romsted L.S., Texter J. (2001). Interfacial Compositions of Surfactant Assemblies by Chemical Trapping with Arenediazonium Ions: Method and Applications. Reactions and Synthesis in Surfactant Systems.

[84] Losada-Barreiro S., Bravo Díaz C., Paiva Martins F., Romsted L.S. (2013). Maxima in antioxidant distributions and efficiencies with increasing hydrophobicity of gallic acid and its alkyl esters. The pseudophase model interpretation of the ‘‘cut-off’’ effect. J. Agric. Food Chem..

[85] Gao X., Bravo-Díaz C., Romsted L.S. (2013). Interpreting ion-specific effects on the reduction of an arenediazonium Ion by *t*-butylhydroquinone (TBHQ) using the pseudophase kinetic model in emulsions prepared with a zwitterionic sulfobetaine surfactant. Langmuir.

[86] Lisete-Torres P., Losada-Barreiro S., Albuquerque H., Sánchez-Paz V., Paiva-Martins F., Bravo-Díaz C. (2012). Distribution of hydroxytyrosol and hydroxytyrosol acetate in olive oil emulsions and their antioxidant efficiency. J. Agric. Food Chem..

[87] Gu Q., Bravo-Díaz C., Romsted L.S. (2012). Using the pseudophase kinetic model to interpret chemical reactivity in ionic emulsions: Determining antioxidant partition constants and interfacial rate constants. J. Colloid Interface Sci..

[88] Chauduri A., Loughlin J.A., Romsted L.S., Yao J. (1993). Arenediazonium Salts: New Probes of Interfacial Compositions of Association Colloids. 1. Basic Approach, Methods and Ilustrative Applications. J. Am. Chem. Soc..

[89] Chauduri A., Romsted L.S., Yao J. (1993). Arenediazonium Salts: New Probes of Interfacial Compositions of Association Colloids. 2. Binding Constants of Butanol and Hexanol in Aqueous Three-Component Cetyltrimehylamonium Bromide Microemulsions. J. Am. Chem. Soc..

[90] Freiría-Gándara J., Losada-Barreiro S., Paiva-Martins F., Bravo-Díaz C. (2018). Differential Partitioning of Bioantioxidants in Edible Oil–Water and Octanol–Water Systems: Linear Free Energy Relationships. J. Chem. Eng. Data.

[91] Figueroa-Espinoza M.C., Villeneuve P. (2005). Phenolic acids enzymatic lipophilization. J. Agric. Food Chem..

[92] Kahveci D., Laguerre M., Villeneuve P. (2015). Phenolipids as New Antioxidants: Production, Activity, and Potential Applications. Polar Lipids.

[93] Romsted L.S., Zhang J. (2002). Kinetic method for Determining Antioxidant Distributions in Model Food Emulsions: Distribution constants of TBHQ in Mixtures of Octane, Water and a Nonionic Emulsifier. J. Agric. Food. Chem..

[94] Gunaseelan K., Romsted L.S., González-Romero E., Bravo-Díaz C. (2004). Determining Partition Constants of Polar Organic Molecules between the Oil/Interfacial and Water/ Interfacial Regions in Emulsions: A Combined Electrochemical and Spectrometric Method. Langmuir.

[95] Sánchez-Paz V., Pastoriza-Gallego M.J., Losada-Barreiro S., Bravo-Diaz C., Gunaseelan K., Romsted L.S. (2008). Quantitative determination of a-tocopherol distribution in a tributyrin/Brij 30/water model food emulsion. J. Colloid Interface Sci..

[96] Costa M., Losada-Barreiro S., Paiva-Martins F., Bravo-Díaz C., Romsted L.S. (2015). A direct correlation between the antioxidant efficiencies of caffeic acid and its alkyl esters and their concentrations in the interfacial region of olive oil emulsions. The pseudophase model interpretation of the ‘‘cut-off’’ effect. Food Chem..

[97] Losada-Barreiro S., Sánchez Paz V., Bravo Díaz C. (2012). Effects of emulsifier hydrophile–lipophile balance and emulsifier concentration the distributions of gallic acid, propyl gallate, and a-tocopherol in corn oil emulsions. J. Colloid Interface Sci..

[98] Freiría-Gándara J., Losada-Barreiro S., Paiva-Martins F., Bravo-Díaz C. (2018). Enhancement of the antioxidant efficiency of gallic acid derivatives in intact fish oil-in-water emulsions through optimization of their interfacial concentrations. Food Funct..

[99] Pastoriza-Gallego M.J., Losada-Barreiro S., Bravo-Díaz C. (2015). Interfacial kinetics in octane based emulsions. Effects of surfactant concentration on the reaction between 16-ArN2+ and octyl and lauryl gallates. Colloids Surf. A Physicochem. Eng. Asp..

[100] Savelli G., Germani R., Brinchi L., Texter J. (2001). Reactivity control by Aqueous self-Assembling Systems. Reactions and Synthesis in Surfactant Systems.

[101] Oehlke K., Heins A., Stöckmann H., Schwarz K. (2010). Impact of emulsifier microenvironments on acid—Base equilibrium and activity of antioxidants. Food Chem..

[102] Fendler J.H., Fendler E.F. (1975). Catalysis in Micellar and Macromolecular Systems.

[103] Romsted L.S. (1985). Quantitative Treatment of Benzimidazole Deprotonation Equilibria in Aqueous Micellar Solutions of Cetyltrimetylammonium Ions (CTAX, X = Cl^−^, Br^−^ and NO_3_^−^) Surfactants. J. Phys. Chem..

[104] Romsted L.S. (2007). Do Amphiphile Aggregate Morphologies and Interfacial Compositions Depend Primarily on Interfacial Hydration and Ion-Specific Interactions? The Evidence from Chemical Trapping. Langmuir.

[105] Quina F.H., Politi M.J., Cuccovia I.M., Martins-Franchetti S.M., Chaimovich H., Mittal K.L., Fendler E.J. (1982). Alkaline hydrolysis in micellar sodium dodecyl sulfate: The binding of hydroxide ion to anionic micelles. Solution Behavior of Surfactants: Theoretical and Applied Aspects.

[106] Bravo Díaz C., Rappoport Z., Liebman J.F. (2011). Diazohydroxides, diazoethers and related species. The Chemistry of Hydroxylamines, Oximes and Hydroxamic Acids.

[107] Maskill H. (1995). The Physical Basis of Organic Chemistry.

[108] Martinez-Aranda N., Losada-Barreiro N., Bravo-Díaz C., Romsted L.S. (2014). Influence of Temperature on the Distribution of Catechin in Corn oil-in-water Emulsions and some Relevant Thermodynamic Parameters. Food Biophys..

[109] Losada-Barreiro S., Sánchez Paz V., Bravo Díaz C., Paiva Martins F., Romsted L.S. (2012). Temperature and emulsifier concentration effects on gallic acid distribution in a model food emulsion. J. Colloid Interface Sci..

[110] Turro N.J., Yekta A. (1978). Luminescent probes for detergent solutions. A simple procedure for determination of the mean aggregation number of micelles. J. Am. Chem. Soc..

[111] Bunton C.A., Savelli G. (1986). Organic Reactivity in Aqueous Micelles and Similar Assemblies. Adv. Phys. Org. Chem..

[112] Biasutti M.A., Abuin E., Silber J., Correa M., Lissi E.A. (2008). Kinetics of reactions catalyzed by enzymes in solutions of surfactants. Adv. Colloid Interface Sci..

[113] López-Cornejo P., Pérez P., García F., de la Vega R., Sánchez F. (2002). Use of the Pseudophase Model in the Interpretation of Reactivity under Restricted Geometry Conditions. An Application to the Study of the [Ru(NH3)5pz]2+ + S2O82- Electron-Transfer Reaction in Different Microheterogeneous Systems. J. Am. Chem. Soc..

[114] Benjamin I. (1996). Chemical Reactions and Solvation at Liquid Interfaces: A Microscopic Perspective. Chem. Rev..

[115] Bondar A.-N., Lemieux M.J. (2019). Reactions at Biomembrane Interfaces. Chem. Rev..

[116] Zarbin A.J.G. (2021). Liquid–liquid interfaces: A unique and advantageous environment to prepare and process thin films of complex materials. Mater. Horizons.

